# Nutritional composition of honey bee food stores vary with floral composition

**DOI:** 10.1007/s00442-017-3968-3

**Published:** 2017-10-14

**Authors:** Philip Donkersley, Glenn Rhodes, Roger W. Pickup, Kevin C. Jones, Eileen F. Power, Geraldine A. Wright, Kenneth Wilson

**Affiliations:** 1 Present Address: Lancaster Environment Centre, Lancaster University, Lancaster, LA1 4YQ UK; 2 Lake Ecosystems Group, Centre for Ecology and Hydrology, Lancaster, LA1 4AP UK; 3 Division of Biomedical and Life Sciences, Lancaster University, Lancaster, LA1 4YQ UK; 4 Institute of Neuroscience, Newcastle University, Medical School, Newcastle upon Tyne, NE2 4HH UK

**Keywords:** Pollen, Pollinators, Diet, Floral community, Amino acids

## Abstract

Sufficiently diverse and abundant resources are essential for generalist consumers, and form an important part of a suite of conservation strategies for pollinators. Honey bees are generalist foragers and are dependent on diverse forage to adequately meet their nutritional needs. Through analysis of stored pollen (bee bread) samples obtained from 26 honey bee (*Apis mellifera* L.) hives across NW-England, we quantified bee bread nutritional content and the plant species that produced these stores from pollen. Protein was the most abundant nutrient by mass (63%), followed by carbohydrates (26%). Protein and lipid content (but not carbohydrate) contributed significantly to ordinations of floral diversity, linking dietary quality with forage composition. DNA sequencing of the ITS2 region of the nuclear ribosomal DNA gene identified pollen from 89 distinct plant genera, with each bee bread sample containing between 6 and 35 pollen types. Dominant genera included dandelion (*Taraxacum*), which was positively correlated with bee bread protein content, and cherry (*Prunus*), which was negatively correlated with the amount of protein. In addition, proportions of amino acids (e.g. histidine and valine) varied as a function of floral species composition. These results also quantify the effects of individual plant genera on the nutrition of honey bees. We conclude that pollens of different plants act synergistically to influence host nutrition; the pollen diversity of bee bread is linked to its nutrient content. Diverse environments compensate for the loss of individual forage plants, and diversity loss may, therefore, destabilize consumer communities due to restricted access to alternative resources.

**Electronic supplementary material:**

The online version of this article (10.1007/s00442-017-3968-3) contains supplementary material, which is available to authorized users.

## Introduction

Biodiversity is central to the sustainable functioning of ecosystems. Complex landscapes enhance resource diversity, which can be better exploited by their inhabitants (Duffy et al. 2007). Resource diversity can result in increased consumer community diversity and broader ecosystem function (Balvanera et al. 2006; De Deyn et al. 2004). Recently studies have begun to investigate the effects of resource diversity at the individual level (Drescher et al. 2014). Three distinct mechanisms explain how biodiversity can result in benefits to a community—through redundancy (where resource scarcity in one species is compensated by another), complementarity (where diverse diets have direct benefits to consumer growth, development or immune function) and “functional balance” (where multiple sources of resource enable balancing of intake to a target) (Drescher et al. 2014; Finke and Snyder 2008; Wohl et al. 2004).

Although organisms regulate their nutritional intake towards an intake target, generalist and specialist consumers have distinct responses to environmental biodiversity. When offered nutritionally complimentary foods, a nutrition generalist such as the desert locust (*Schistocerca gregaria* L.) will consumer a greater excess of more abundant macronutrients and a smaller deficit of limiting nutrients. In contrast, nutritional specialists, such as the African migratory locust (*Locusta migratoria* L.), cannot benefit from dietary diversity and consequently suffer a substantial deficit in limiting nutrients to avoid over-consuming the excess nutrient (Raubenheimer and Simpson 2003).

Although some pollinators, such as some moths and solitary bees, can be specialist consumers, typically generalists are more effective pollinators as they can pollinate many flowering plant assemblages through the year (Motten et al. 1981). Consequently, they benefit distinctly from more diverse environments. Amongst consumer communities, bees are unique. In addition to selectively feeding on nectar and pollen provided by flowering plants, they aid these plants with reproduction through pollination. Evidence suggests pollinators benefit from resource complementarity (Alaux et al. 2010) and the plant communities benefit from redundancy, where if one pollinator goes extinct, a plant can still be pollinated by another species (Blüthgen and Klein 2011). The reciprocal benefit means that as plant communities grow more diverse through the transport of genetic material by pollinators (Woodcock et al. 2013), in turn these communities become more capable of supporting their pollinators (Blüthgen and Klein 2011).

Widespread declines of insect pollinators have occurred across much of Europe since the start of the millennium (Potts et al. 2010); declines in this reciprocal system indicate systemic problems impacting pollination. Evidence suggests this may be occurring through a combination of agricultural intensification, habitat degradation and the spread of pests and pathogens (Goulson et al. 2015). These losses are threatening pollination services, and therefore, food security (Steffan-Dewenter et al. 2005). To stem the decline of bees, planting schemes to provide floral resources for bees have been developed amongst a suite of methods, also including protection of nesting sites and control of agrochemical applications to enhance pollinator resilience and help prevent further decline (Decourtye et al. 2010; Scheper et al. 2013).

Agricultural habitats are arguably where pollinators are most important, yet these are sites of significant pressure on pollinators (Deguines et al. 2014). Agricultural habitats provide a huge source of floral resources over short periods, but agricultural intensification impacts habitat diversity and availability at a local scale (Holzschuh 2016). Small numbers of wildflowers and trees, such as *Acer* spp., *Prunus* spp., or *Salix* spp., provide particularly attractive forage resources (Requier et al. 2015). Evidence suggests that wildflowers are preferentially visited by both honey and bumble bees over more abundant crop flowers, such as *Helianthus annuus* or *Brassica napus* (Kämper et al. 2016; Requier et al. 2015). Furthermore, foraging by pollinators is demonstrably affected by the land use composition (Kleijn and van Langevelde 2006; Klein et al. 2007). The nutrition that pollinators can derive is clearly linked to their environment (Donkersley et al. 2014), yet this link has not been investigated in terms of forage composition.

The European honey bee (*Apis mellifera* L.) is an important pollinator of agricultural systems around the world and is becoming one of several model systems for studying the causes and consequences of pollinator declines (Calderone 2012). Honey bees forage for pollen and nectar as their primary source of nutrition. When storing in the hive, pollen is mixed with nectar as a material known as “bee bread” (Herbert and Shimanuki 1978). Although the nature of fresh pollen collected at the entrance of the hive is well documented (Dimou and Thrasyvoulou 2009; Keller et al. 2015; Kleijn and Raemakers 2008), evidence suggests pollen stored within the hive may also be a key part of the diet (Anderson et al. 2014). Yet, this material is comparatively poorly understood (Donkersley et al. 2014; Foulis and Goulson 2014) and is, therefore, the focus of this study.

Quantitative identification of pollen is the key for studying the link between diet and forage in pollinators (Richardson et al. 2015a). Traditionally, bee foraging behaviour has been assessed in one of two ways: pollen traps, designed to remove grains of pollen from the legs of forager bees entering the hive (Koppler et al. 2007) and observation of bees foraging on plants in the field either directly (Haaland et al. 2011) or through harmonic radar tracking (Osborne et al. 1999). Pollen analysis has been accomplished using microscopic palynology, a technique involving the discrimination of pollen types by morphology (Ohe et al. 2004). Due to the expertise required and difficulties associated with accurately distinguished pollen, this technique has been difficult to implement on a large scale (but see Martin and Harvey 2017). Molecular fingerprinting methods that target plant DNA allow detection and identification of the species assemblage of pollen inside the bee hive (Keller et al. 2015). Although this method has been successful for identification of monospecific pollen (Matsuki et al. 2008; Suyama 2011), recently next-generation sequencing was used to characterize the botanical origins of bee-collected pollen (Richardson et al. 2015b). The results of sequencing studies correlate with microscopic palynology (Keller et al. 2015), but both methods are limited by the availability of voucher specimens for identification (Richardson et al. 2015b). Further, both methods have issues with accurate quantification of pollen grains: molecular methods may require a multi-locus approach due to variation in gene copy number between species (Richardson et al. 2015a), microscopic palynology relies on a subsample of the total sample and may overlook low abundance pollens (Ohe et al. 2004).

The most important components of bee nutrition are proteins, carbohydrates, lipids and amino acids, with each having significant impacts upon individual fitness (Paoli et al. 2014; Vanderplanck et al. 2014; Vaudo et al. 2015); following Vanderplanck (2014), we refer to the combined benefits of a high quality bee bread in terms of all these factors as “nutritional value”. The protein, carbohydrate, lipid and amino acid contents (nutritional value) of pollen vary across species (Roulston and Cane 2000). Resource diversity implies that organisms can optimize the composition of nutritional resources through complementarity (Drescher et al. 2014).

In this study, we therefore, aimed to test two hypotheses: the first that the nutritional value of stored pollen (bee bread) will be linked to its pollen community composition; that through resource complementarity, more diverse environments provide improved nutrition. Second, we hypothesise that through resource redundancy, when a dominant pollen species is lost, no significant impact will be seen on the dietary nutritional content. We therefore, present a DNA fingerprinting study of plant species found in bee bread and an analysis of the nutritional composition of these samples in terms of proteins, amino acids, and carbohydrates to test these hypotheses.

## Methods

### Bee bread sampling

Fifty-one samples of bee bread, comprising individual cells from unique frames, were collected from 26 European honey bee (*A. mellifera* subsp. *mellifera*) hives within 19 apiary sites in north-west England between 7th April and 2nd September 2012 (Table S1). These samples were a subset from a previous study, due to constraints in the scope of this study. The subset was chosen to be the most representative of the spectrum of nutritional contents (protein and carbohydrate) determined in the previous study (Donkersley et al. 2014), based on data points distributed around the mean, variance and min/max values of the original data set.

The hives were owned by either hobbyist beekeepers, or maintained as part of training suites for local beekeeping associations. To minimize contamination of the samples, each cell was extracted using a separate, sterile sampling tool and placed into sterile 1.5 ml microfuge tubes for transport to the laboratory. Samples were returned to the lab and stored within 2 h of collection. The mean mass (± S.D.) of bee bread samples was 130.12 mg ± 62.97; these samples were homogenised using a micropestle and divided for nutritional and molecular analysis, with 85% of the sample (111.40 mg ± 52.91) being used for nutritional analysis and the remaining 15% (19.70 mg ± 9.34) for DNA extraction and amplification (Table S1).

### Macronutrient analysis

For protein, carbohydrate and lipid assays, absorbance was measured using a VERSAmax™ Tunable Microplate Reader (Molecular Devices, Sunnyvale, CA) set to 550, 575, 510 and 540 nm, respectively, using Softmax^®^ Pro v4.7 software for Windows^®^. Protein was estimated using the Biuret reaction standardised against a bovine serum albumin dilution series (Sapan et al. 1999); carbohydrate using the dinitrosalicylic acid (DNS) reaction using glucose and sucrose as standards (Miller 1959); lipid using phosphoric acid–vanillin analysis colorimetry, with sunflower oil as a standard (Cheng et al. 2011). Water content of bee bread samples was determined by placing bee bread in a drying oven at 100 °C for 24 h and calculating the difference in mass between wet and dried samples. Dietary preferences and host fitness in insects often correlates with dietary protein:carbohydrate (Simpson et al. 2015) and protein:lipid ratios (Vaudo et al. 2015), we therefore, estimated these using protein/carbohydrate and protein/lipid for each sample.

### Amino acid analysis

Amino acid composition of bee bread was analysed using ultra-high-performance liquid chromatography (uHPLC). The mass of bee bread used for extraction was 3.31 mg ± 2.51. Free amino acids were extracted first: each sample was placed in 300 μl HPLC-grade methanol (Sigma-Aldrich, Dorset, UK.) and mixed for 60 s in an electrical vortex to extract free amino acids, followed by centrifugation at 13,000 rpm for 5 min. The supernatant was filtered through a 0.45 µm syringe-tip filter (Whatman Puradisc 4, nylon 4 mm) to remove particulates and was then analysed for free amino acids. The remaining pellet was analysed for protein-bound amino acids using the methods described in Stabler et al. (2015). Briefly, the pellet was dried down at 70 °C; mixed with 30 µl 6 M hydrochloric acid (HCl) and the sample was briefly vortexed. Sealed tubes were placed in plastic microfuge tube boxes, sealed, and placed in a domestic 900 W (2450 MHz) microwave oven inside of a fume hood according to Zhong et al. (2005). Samples were irradiated for 15 min on full power, left to cool, and then heated at 70 °C in a heat block to evaporate the acid. Once dry, 300 µl of de-ionised uHPLC gradient grade water was added to each sample, centrifuged for 1 min and filtered through 0.45 µm syringe-tip filters (Whatman Puradisc 4, nylon, 4 mm). Ten µl of each filtered sample was analysed using the uHPLC. We quantified 21 amino acids in the samples using a Dionex Ultimate 3000 RS system fitted with a 150 × 2.1 mm Accucore RP-MS (Thermo Scientific) column using methods described in Stabler et al. (2015). Amino acids present in 10 μl of a 1:500 dilution of the bee bread extracts were identified and quantified by comparison with Sigma-Aldrich AA-S-18 amino acid calibration standards supplemented with asparagine, glutamine tryptophan, and γ-aminobutyric acid (GABA), diluted to 2.5 μM using HPLC-grade water.

### PCR amplification

DNA was extracted from each of the 51 bee bread samples using the QIAamp DNeasy Plant Mini kit (Qiagen Ltd, Crawley, UK). DNA extractions were performed according to manufacturers’ specifications. The ITS2 region of the nuclear ribosomal DNA gene was selected as a barcode to estimate plant diversity in bee pollen forage within the hive (Chen et al. 2010). PCR amplification of ITS2 was performed using primers SF2 and S3R (Table S2) with an 8 bp sample specific index sequence on the forward primer for each sample (i.e. GACATAAT–TAATGCCA). PCR cycling conditions were as follows: initial denaturation at 94 °C for 3 min, followed by 28 cycles of 94 °C for 30 s, 53 °C for 40 s and 72 °C for 60 s, with a final elongation step at 72 °C for 5 min. PCR products were quantified using a spectrophotometer (NanoDrop ND-1000, ND Technologies, Wilmington, DE) and pooled equally based on DNA concentration. PCR products were visualized after agarose gel electrophoresis. Amplified products were then purified using calibrated Ampure XP beads (Beckman Coulter, USA).

### DNA fingerprinting and data processing

PCR amplification products were sequenced using a commercial facility at Molecular Research LP (http://www.mrdnalab.com, Shallowater, TX, USA) on the Illumina MiSeq platform using Illumina TruSeq DNA library preparation protocol for 2 × 300 bp paired-end reads following the manufacturer’s guidelines, with 24 samples per lane.

Sequences were first filtered by Phred quality scores using a standard Q25 20 bp window. Data processing was then performed in Mothur v. 1.36.1 (Schloss et al. 2009), using the MiSeq SOP (Kozich et al. 2013). Briefly, paired-end sequences were merged using “make.contigs”, sequences with ambiguous bases or shorter than 450 bp were removed with “screen.seqs” and chimeric sequences were removed using “chimera.uchime”. Sequences were clustered into operational taxonomic units (OTUs) using the “dist.seqs” and “cluster” functions. Alignment was performed using the BLASTn algorithm (Altschul et al. 1990), with quality thresholds set as: *E* value cutoff 1 × 10^−150^, single alignment only, percent identity threshold 95% (Richardson et al. 2015b) following the MiSeq SOP (Kozich et al. 2013). Final OTUs were classified using the “classify.otu” function against a curated international database of ITS2 sequences matching the taxonomic group Viridiplantae compiled from Ribosomal Database Project RDPII (https://rdp.cme.msu.edu), NCBI SRA (http://www.ncbi.nlm.nih.gov) and GreenGenes (v13.5, http://greengenes.lbl.gov/), assigning genus identity to reference sequences (DeSantis et al. 2006).

### Statistical analysis

Analyses were performed within the R statistical software v3.3.2 (2016). Community composition was first characterised into diversity indices (Shannon and evenness) using the ‘Vegan’ package (Oksanen et al. 2013). The Shannon index describes species equitability as a function of total diversity, and evenness ranges from 0 (only one species sampled) to 1 (all species being equally sampled).

As rarefaction of metagenomic data may increase type-II errors (McMurdie and Holmes 2014), we instead normalised the data using the “normFactor” function in the ‘metagenomeSeq’ package for R (Paulson et al. 2016). Instead of removing results as with rarefaction, normalisation calculates a factor to equalise results between samples. OTU count data were modified by sample specific normalisation factors and used for downstream analysis.

Recent studies of sequencing data of bee bread indicate that OTU counts of pollen species are in some cases correlated to microscopic pollen grain counts (Keller et al. 2015). However, without validation using other sequencing regions, statistical analysis of abundance data is of uncertain value (Richardson et al. 2015a). Non-metric multidimensional scaling (NMDS) allows for analysis of communities based on variation in the abundances of all members of the community (Wang et al. 2012). Here, we analysed community count data by NMDS using the “metaMDS” function (Oksanen et al. 2013). NMDS was performed using the Bray–Curtis dissimilarity index on three ordinal scales for optimal NMDS stress values. NMDS results were used as a parameter for total plant community composition, directional cosines (the contribution of a variable to the “slope” of a vector) between each NMDS vector and nutritional contents were tested and assigned significance using the “envfit” function. Nutritional contents tested included: protein (*P*), carbohydrate (*C*), lipid (*L*), moisture and both *P*:*C* and *P*:*L* ratios.

Effects of diversity indices and the most common individual plant genera (that accounted for 95% of sequence reads; dependent variables) on bee bread nutritional content (independent variables) were analysed. Due to the semi-nested design resulting from using a data subset, we also tested for random effects of hive identity and sample month using linear mixed effects models (LMER) with log-transformed continuous dependent variable, using residual maximum likelihood (“rand” function) testing for each of these genera models (Zuur, 2009). Bonferroni critical P scaling (Benjamini and Hochberg 1995) was used to reduce the probability of type-I errors in these models, which resulted in a revised critical *P* of 0.03 for these analyses. For comparison with latter data, post hoc correlations for significant models were calculated.

The concentrations of amino acids in bee bread, the relative amino acid to sugar ratio ($\sum {{\text{aminos}}/{\text{carb}}}$) and amino to lipid ratio ($\sum {{\text{aminos}}/{\text{lipid}}}$) were analysed using Spearman’s partial correlations with individual genus abundance (Fig. 2) using the “cor” function in the ‘fields’ package in R (Nychka et al. 2014). To reduce the probability of type-I errors in these correlations, critical *P* value was set to 0.01.

## Results

### Macronutrient content of bee bread

**Fig. 1:**
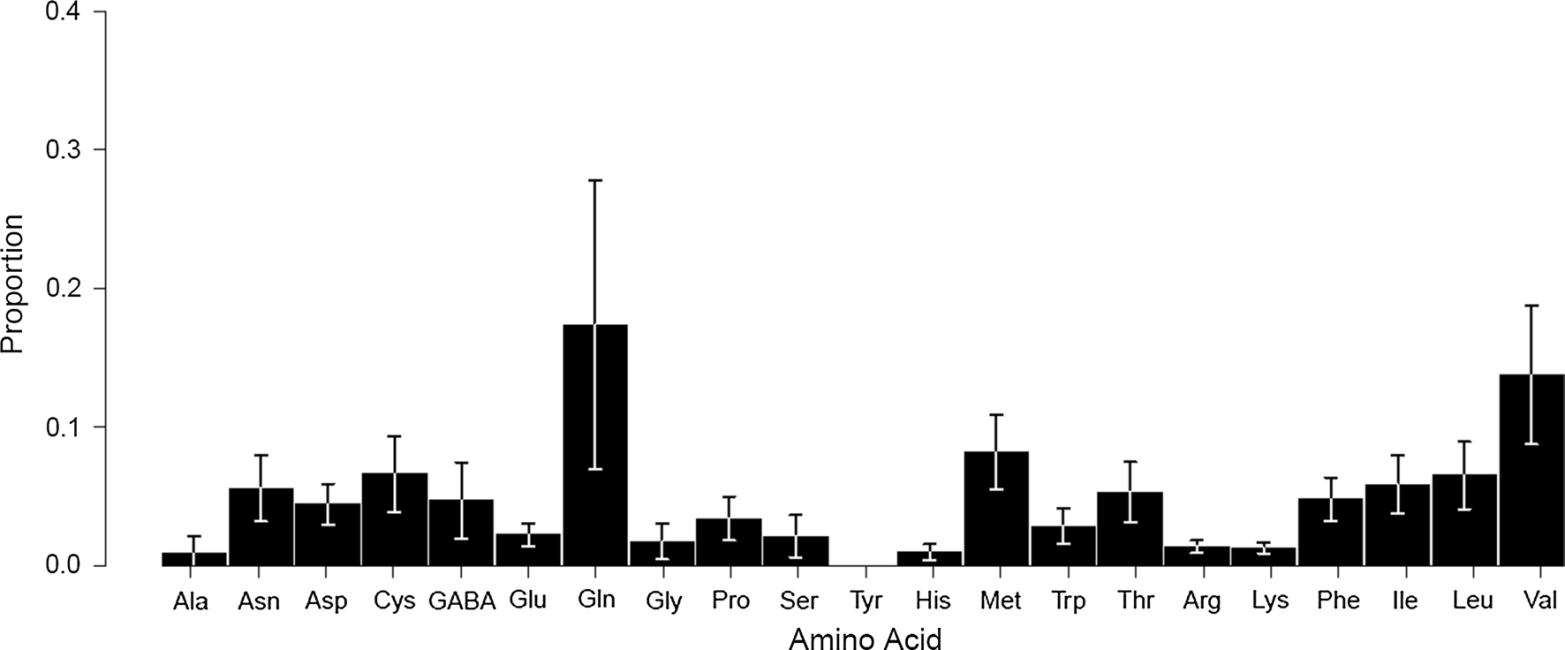
Mean proportions of amino acids of the total amino acid content from across all samples. *Asp* aspartate, *Glu* glutamate, *Asn* asparagine, *Ser* serine, *Gln* glutamine, *His* histidine, *Gly* glycine, *Thr* threonine, *Arg* arginine, *Ala* alanine, *GABA* γ-aminobutyric acid, *Tyr* tyrosine, *Cys* cysteine, *Val* valine, *Met* methionine, *Trp* tryptophan, *Phe* phenylalanine, *Ile* isoleucine, *Leu* leucine, *Lys* lysine, *Pro* proline. Bars represent standard deviation of the mean proportion

The major nutritional constituent of the bee bread was protein (mean ± SD = 629 mg g^−1^ ± 290 wet weight), followed by reducing sugars (130 mg g^−1^ ± 63) and non-reducing sugars (119 mg g^−1^ ± 85). Lipids and starch were present in low concentrations (38 ± 2 and 13 ± 8 mg g^−1^, respectively). The mean water content was 29% (290 ± 180 mg g^−1^). The protein to carbohydrate ratio was 1.58:1 ± 1.00. µHPLC analysis revealed 17 amino acids present in the 49 bee bread samples (2 samples were lost during HPLC analysis; Fig. 1, Table S4). Among those amino acids detected, glycine accounted for the greatest proportion of total amino acids (mean ± SD = 0.17 ± 0.13); other dominant amino acids included valine (0.14 ± 0.04) and methionine (0.08 ± 0.01).

### Diversity of plant species in bee bread

Sequencing of amplified DNA from bee bread generated 5,655,523 raw reads with an average read length of 470 bp (456–488 bp) clustered into 1388 distinct operational taxonomic units (OTUs). Post filtering, 403 OTUs, accounting for 682,747 reads, were disregarded due to low identity and *E* value scores leaving a total of 4,972,776 reads for sequence alignment. Sequences derived from this study were deposited on the NCBI Sequence Read Archive (http://trace.ncbi.nlm.nih.gov/Traces/sra) under accession numbers SRR1612417 and SRR1612418.

Following sequence alignment, the OTUs were classified to 44 families, 89 distinct genera (Figure S1) with each sample of bee bread containing an average of 29 different plant genera (mean ± SD 28.25 ± 9.86, range 6–35). The average Shannon diversity index (or equitability) generated from profiles was 0.99 (± 0.41, range 0.31–2.01) and evenness was 0.42 (± 0.20, range 0.07–0.54), indicating that overall, samples were mostly dominated by a small number of very abundant genera. The five most common genera across all samples were *Trifolium* (reads 973410; mean ± SD 11.80% ± 22.94), *Impatiens* (575888; 8.42% ± 22.62), *Rubus* (551618; 8.39% ± 19.59), *Acer* (496974; 11.82% ± 28.19) and *Cirsium* (450344; 3.68% ± 16.78; Figure S1). These five genera occurred in up to > 90% (47/51) of the bee bread samples, with *Trifolium* being detected in all samples.

Furthermore, 16 genera accounted for > 95% of the total number of sequence reads in our survey. In order of abundance these were *Trifolium*, *Impatiens*, *Rubus*, *Acer*, *Cirsium*, *Euscaphis*, *Cryptotaenia*, *Glycine*, *Coriandrum*, *Rosa*, *Prunus*, *Taraxacum*, *Camelina*, *Ranunculus*, *Salix* and *Andira* (Figure S1). Within some samples, *Impatiens* sequences were the most dominant, accounting for up to 91% of sequences.

### Bee bread macronutrient content correlates with its pollen diversity and composition

**Fig. 2:**
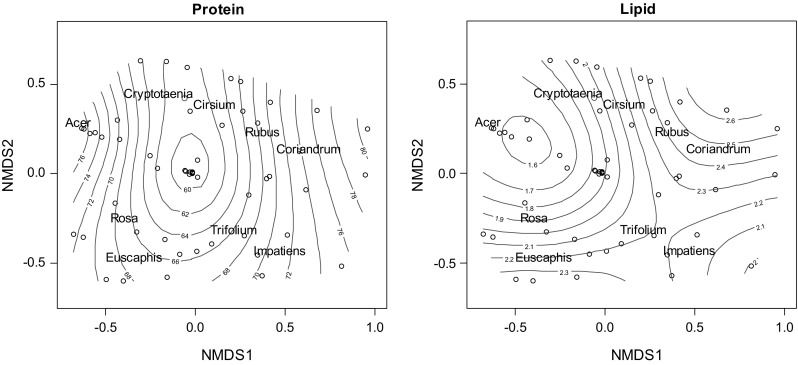
Surface NMDS ordinations of bee bread plant communities from 51 bee breads, denoted by open circles, with their position determined by where they fall on ordinal axes 1 and 2. Red names are species centroids from these communities indicating bee bread samples dominated by these species; blue contour lines indicate corresponding nutrient contents that correlated with ordinal axes, which are interpreted as how each species in the community (and the overall community composition) correlate with the nutritional contents of bee bread (colour figure online)

**N**MDS showed that a three-dimensional solution was sufficient to achieve low stress values to enable us to interpret plant community composition (stress = 0.14). Macronutrient composition of bee bread was significantly correlated with the NMDS analysis of bee bread pollen composition (Table 1; Fig. 2). The protein content of bee bread contributed significantly to ordinal vectors 1 and 3 (*r* ^2^ = 0.17, *P* = 0.03); lipid content also contributed significantly to ordinations 1 and 2 (*r* ^2^ = 0.19, *P* = 0.02; Table 1). For example, higher protein content bee breads were correlated with communities dominated by *Acer*, *Trifolium*, *Impatiens* and *Coriandum* (Fig. 2; Figure S2).

**Table 1:** NMDS correlations with environmental variables and nutritional content

	NMDS1	NMDS2	NMDS3	*r* ^2^ Correlation	*P* value
Moisture	− 0.84	0.04	0.55	0.13	0.09
Lipid	0.56	− 0.48	0.68	0.18	0.03
Protein	0.10	0.50	− 0.86	0.20	0.02
Carbohydrate	0.19	0.79	− 0.58	0.10	0.15
*P*:*C* ratio	− 0.26	0.19	− 0.95	0.07	0.36
*P*:*L* ratio	− 0.19	0.48	− 0.85	0.05	0.36

Centroids for each of the variables against three-dimensional ordinations of plant community calculated using NMDS, correlations and *P* values are calculated though the “envfit” function in R

The abundances of some individual plant genera (OTUs) were correlated with the macronutrient contents of bee bread. Although the most common species were not correlated with nutritional content (i.e. *Trifolium, Impatiens, Rubus*, etc.); after controlling for the probability of type-I errors, protein content was negatively correlated with the abundance of *Rosa* (*b* ± SE = − 0.02 ± 0.01, *F*_1,50_ = 5.38, *P* = 0.03; *posthoc R* ^2^ = − 0.31) and *Prunus* (b ± SE = − 0.03 ± 0.01, *F*_1,49_ = 5.21, *P* = 0.03; *posthoc R* ^2^ = − 0.30) in the bee bread sample. Protein was positively correlated with the abundance of *Taraxacum* (*b* ± SE = 0.36 ± 0.02, F_1, 49_ = 3.69, *P* < 0.01; *posthoc R* ^2^ = 0.26) and *Salix* (*b* ± SE = 0.35 ± 0.01, *F*_1,49_ = 9.42, P < 0.01; *posthoc R* ^2^ = 0.39). *P*:*C* ratio was positively correlated with the abundance of *Prunus* (*b* ± SE = 2.16 ± 0.34, *F*_1,49_ = 5.21, *P* = 0.03; *posthoc R* ^2^ = 0.18). Likelihood ratio tests of the random effects hive and month were all non-significant, indicating that the nature of the data subset did not affect the statistical model structure.

### Bee bread total amino acid composition correlates with its pollen diversity and composition

The amino acid content of bee bread also contributed significantly to ordinations plant diversity (Table 2). For example, amino acid concentrations of histidine (*r* ^2^ = 0.15, *P* = 0.02), lysine (*r* ^2^ = 0.18, *P* < 0.01) and threonine (*r* ^2^ = 0.17, *P* = 0.02) all significantly contributed to the ordinal vectors of plant community composition in bee bread.

**Table 2:** NMDS correlations with environmental variables and amino acid content

	NMDS1	NMDS2	NMDS3	*r* ^2^ correlation	*P* value
Alanine	0.01	1.00	0.35	0.02	0.61
Asparagine,	− 0.21	0.98	0.23	0.04	0.43
Aspartate	0.42	− 0.91	− 0.28	0.02	0.63
Cystine	− 0.07	1.00	0.79	0.03	0.43
γ-Aminobutyric acid	0.44	0.90	0.63	0.05	0.33
Glutamate	− 0.48	0.88	0.48	0.08	0.14
Glutamine	0.81	0.59	0.66	0.04	0.39
Glycine	0.80	0.60	0.56	0.04	0.37
Proline	− 0.13	0.99	0.75	0.04	0.40
Serine	0.61	0.79	0.53	0.12	0.05
Tyrosine	0.10	1.00	0.79	0.05	0.32
Histidine	0.51	0.86	0.59	0.15	0.02
Methionine	− 0.66	0.76	0.85	0.03	0.53
Tryptophan	− 0.13	0.99	0.78	0.04	0.37
Threonine	0.53	0.85	0.19	0.17	0.02
Arginine	0.50	0.87	0.79	0.05	0.27
Lysine	0.46	0.89	0.42	0.18	0.01
Phenylalanine	0.22	0.98	0.63	0.08	0.15
Isoleucine	− 0.04	1.00	0.71	0.07	0.19
Leucine	0.30	0.96	0.66	0.11	0.05
Valine	0.62	0.78	0.16	0.04	0.42

Centroids for each of the variables against three-dimensional ordinations of plant community calculated using NMDS, correlations and *P* values are calculated though the “envfit” function in R

Measures of dietary genus diversity, including alpha diversity (species number) and species evenness were not correlated with total amino acid content (*P* = 0.07 and *P* = 0.22, respectively). However, individual amino acids did correlate with alpha diversity; for example, less diverse diets were found to contain higher histidine levels (Spearman’s rho = − 0.30, *n* = 49, *P* = 0.03).

**Fig. 3:**
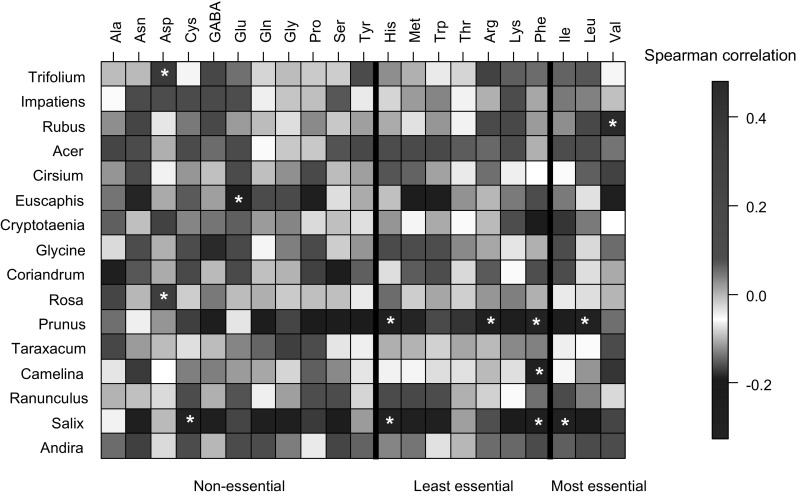
Correlations between amino acid composition and floral diversity as quantified using next generation sequencing. Columns correspond to amino acid concentration (μg/mg bee bread); rows correspond to floral genus abundance accounting for 95% of sequence reads (ordered from most to least abundant genus). Red and blue denote positive and negative association, respectively. The intensity of the colours represents the degree of association between the amino acids and floral species abundances as measured by the Spearman’s correlation (significant correlations occur at |*r*| > 0.333 and are highlighted with asterisks). Essential and non-essential amino acids are separated by borders within the plot (colour figure online)

The abundances of some OTUs were correlated with bee bread amino acid contents, though few significant correlations were detected, even for the more common genera (Fig. 2). *Impatiens* abundance was significantly positively correlated with asparagine (rho = 0.37, *n* = 49, *P* < 0.01), but no other amino acids. Finally, the significant correlations between pollen genera and amino acid content were unidirectional, such that each amino acid was only positive or entirely negatively correlated with each genus (Fig. 3).

## Discussion

This study measured the relationships between the pollen and nutritional compositions of bee bread from hives of the honey bee (*A. mellifera*). The key findings are that this generalist forager benefits from resource complementarity; NMDS demonstrates the nutritional value of bee bread was linked to the composition of pollens that comprise it. These pollens were dominated by 16 genera, including clover (*Trifolium*), balsam (*Impatiens*), blackberry (*Rubus*), sycamore (*Acer*) and thistles (*Cirsium*).

This study used next generation sequencing to identify pollen species in bee bread stores to genus level. Whilst there are many logistical and other advantages to this approach, in presenting our findings we have not overlooked the potential issues associated with these methods. Although next generation sequencing was used to characterize the botanical origins of bee-collected pollen, its capacity to accurately quantify pollen numbers from mono-locus sequencing may be limited (Keller et al. 2015; Richardson et al. 2015b). A multi-locus metabarcoding approach may represent a more accurate method for identifying pollen abundances in a mixed sample (Richardson et al. 2015a); however, this was beyond the scope of the present study.

Through our data processing, we have taken measures to account for biases that may come via PCR-error, sequencing error, sequencing depth, or issues in the bioinformatic pipeline (Lee et al. 2012; Zhou et al. 2011). The processing methods employed here, read length, OTU similarity definition and sequence databases available, currently do not allow for the accurate differentiation between species; therefore, we chose a more conservative approach using genus-level identification. We also note that as bee bread is a mixture of pollen and nectar, and we do not have a method for segregating DNA from these origins from our samples, it is likely that our findings include some nectar-derived sequences (Eady et al. 1995) and therefore, these results may also reflect nectar foraging habits by bees, and not just pollen foraging.

### Floral resources

We observed a total of 44 genetically distinct floral families across 51 bee bread samples in 26 hives, with an average of 29 different genera in each sample. Other studies using the same sequencing technology showed comparable results, Richardson et al. (2015a, b) have shown 45 families within four stored samples using the ITS2 gene, and 49 families across six samples using the ITS2, rcbL and matK genes. Other studies show contrasting results; however, this may be due to differences in sampling and identification methods. Stored pollen and bee bread showed distinct compositions to pollen loads on the bee: sequencing studies of these pollens, using the Roche 454 sequencing technology, detected a lower richness within samples between 4 and 12 genera (Keller et al. 2015). Similarly, lower pollen diversity has been found in studies that use palynological analyses instead of molecular-based approaches: bumble bee colonies in Germany showed low abundances between 6 and 8 pollen morphotypes per colony (Kämper et al. 2016). As well as the sampling method, the sampling program is also important, over a 24 h period, Requier et al. (2015) recorded an average of 15 pollen species per sample.

Within the present study, clover species (*Trifolium*) was the most abundant (greatest proportion of reads) and prevalent (present in all samples) of all the genera found in bee bread (Figure S1). This genus is particularly abundant in improved grasslands and amenity grasslands such as parks and gardens (Carvell et al. 2006; Critchley et al. 2007; Kämper et al. 2016), which were prevalent in the study area (Table S3). Perennial species, particularly in the genus *Acer* and family Rosaceae (e.g. Cherry, Pear or Apple) were found in bee bread, with *Rubus* (5.89%), *Rosa* (4.32%) and *Prunus* (3.89%) being 3rd, 10th and 11th most commonly observed, respectively (Figure S1). These results are consistent with previous studies that show the genus *Acer* and the family Rosaceae are commonly found in bee collected pollens (Kämper et al. 2016; Keller et al. 2015; Richardson et al. 2015a; Requier et al. 2015). We have observed a high variance around the mean abundances of genera in this study, possibly resulting from the social, patch-focused foraging strategies used by honey bees. The samples analysed in this study were from a limited pool of time periods (primarily individual replicates at each time point) and geographical locations (19 sites, see Table S1 and Methods), thus leading us to be unable to accurately analyse the variance statistically.

From the forage genera identified here, of particular import is to note that balsam (*Impatiens*) is the second most common genus (after clover) found in the present study. Within the study area, the most abundant balsam species was the alien invasive Himalayan *Impatiens glandulifera* (P. Donkersley, pers. obs. 2014). Our data indicate that balsam accounts for a significant proportion of the forage collected by bees. What is perhaps more intriguing is that, although it is not present in all samples of bee bread, where it is present, balsam accounts for up to 90% (range = 0.2–91.7%, median = 0.4%) of the sequences, suggesting that this species is particularly attractive to honey bees when it is available, perhaps due to a shortage of alternatives. Himalayan balsam is highly invasive and destructive to riparian ecosystems (Hulme and Bremner 2006). Although it is required to be controlled under Schedule 9 of the UK Wildlife and Countryside Act 1981, options for this are currently limited to mechanical or chemical methods (CEH 2004). Himalayan balsam has been shown to significantly alter pollinator foraging preferences in bees and it can dominate pollen collection by bees in areas where present (Lopezaraiza-Mikel et al. 2007) and is widely considered by beekeepers in our study area as a key resource for both pollen and nectar. Honey bees are generalist foragers, and it is unlikely that the eradication of Himalayan balsam will negatively impact on the UK honey bee population in the long term. Although not definitive, the PCR-based evidence, personal observation and local knowledge of pollen foraging provided by beekeepers suggest that Himalayan Balsam is a key component of the local honey bee diet. This may provide further opportunities for debate on the destructiveness of this plant in riparian habitats and its importance to beekeepers.

Some studies report that legumes are among the most frequently visited plant families by many bee species for pollen (Hanley et al. 2008; Lagerlöf and Wallin 1993). Aside from clover (*Trifolium*), legumes were found in very low abundances in bee bread of honey bees, which has similarly been found for various bumble bee species (Kriesell et al. 2017). Many of the other species of legumes may be in low abundance or missing due to a lack of sufficient forage in the environment. Due to the collective foraging approach employed by honey bees, many preferred food plant flowers may not have been sufficiently abundant to be attractive to forager bees in improved grasslands, based on total nectar or pollen quantity (Fewell and Bertram 1999). As a result, honey bees may rely on non-native floral resources to fulfil nutritional requirements, which are often more locally abundant and possess more visually attractive flowers (Ghazoul 2004).

### Effects of bee bread plant species composition on nutrient content

Bee bread nutritional content was found to vary significantly with some of the most common plant genera (Fig. 2) and with the overall community diversity. The protein content of bee bread was found to correlate with the abundance of specific plant genera (e.g. *Prunus*, *Taraxacum or Salix*). Our study contributes to a growing number of studies on the role of biodiversity on ecosystem functioning (Duffy et al. 2007). Although resource diversity can increase community diversity, resulting in broader ecosystem function (Balvanera et al. 2006; De Deyn et al. 2004), pollinators such as the honey bee illustrate how a single member of that community can benefit from resource diversity (Drescher et al. 2014). Through NMDS we demonstrated that bee bread “nutritional value” contributes significantly to the ordinations of pollen diversity, suggesting functional complementarity; complex communities are linked to increasing protein content (Yachi and Loreau 2007). Previous studies have quantified the benefits of dietary diversity on bee fitness; a direct benefit to honey bee immunity occurs when they are fed a species diverse diet (Alaux et al. 2010).

Nutrition-derived fitness is often linked to a balance of both protein:carbohydrate ratios and amino acids (Simpson et al. 2015). The effects of certain members of the forage community on “nutritional value” also suggests that pollinators may benefit from “functional balance”, where multiple sources of different nutritional contents enable the balancing of intake to a target (Drescher et al. 2014; Finke and Snyder 2008; Wohl et al. 2004). We have previously demonstrated variation in bee bread “nutritional value” within hives at inter-frame and inter-box levels and suggested this is due to clustering of tasks by groups of bees within the hive (Donkersley et al. 2014). Based on this and research by Kriesell et al. (2017), we suggest that achieving the intake target for macronutrients and amino acids within the hive may be accomplished by the mixing of multiple bee breads of differing nutritional contents. Similarly, previous studies with bumble bees suggest that the availability of particular plants, rather than total plant diversity, is a major driving factor in how bees secure nutrient-rich diets (Kämper et al. 2016).

Honey bees do not forage randomly and their behaviour is modulated by learning which plants have an optimum “nutritive value” (Hendriksma and Shafir 2016). Foraging preferences are also influenced by the addictive qualities and visual attractiveness of plants, which are not always linked to nutritional rewards (Nicholls and Hempel de Ibarra 2017; Thomson et al. 2015). As such, it is important to consider both the species composition of these food stores and their nutritional content. Although floral resources may be sufficiently abundant and attractive, they can support healthy bee populations only when they contain sufficient nutritional macronutrients (protein, carbohydrate, lipid, etc) and micronutrients (amino acids, vitamin, minerals, etc) (Avni et al. 2014; Hendriksma et al. 2014).

The results presented here also account for the variable amino acid contents of bee bread and how this influences overall bee bread amino acid content. All of the essential amino acids suggested by deGroot (1953) for optimal development and survival of caged honey bee colonies, were detected in all of the bee bread samples, indicating that the current mixtures of pollen that honey bees are collecting within the study site may be sufficient to meet their amino acid requirements and/or that honey bees are selecting pollen that contains the amino acid profiles they require. Here, in terms of individual amino acids, we found only unidirectional correlations with particular genera, meaning that the abundance of a given plant (e.g. *Trifolium* or *Rubus*) has only positive significant correlations with amino acids, whereas other genera (e.g. *Prunus* or *Salix*) have only negative correlations, suggesting these latter forage species may have diluting effects to dietary amino acid content when present in sufficient quantities.

The factors that drive temporal trends in pollinator foraging dynamics are numerous and complex; including plant resource availability, landscape diversity and climate on the broad scale and weather patterns on the local scale (Decourtye et al. 2010; Kaluza et al. 2016). Given the benefits of resource diversity, a general lack thereof found in environments with reduced biodiversity may destabilize consumer populations due to reduced compensatory and/or synergistic mechanisms. This effect can be observed with the decline of pollinators in Europe associated with agricultural intensification and habitat degradation (Potts et al. 2010). The link between “nutritional value” and forage composition potentially provides an important relationship between environmental biodiversity (i.e. land use) and functional synergisms, which may play a role in pollinator decline (Drescher et al. 2014).

### Land use and environmental context

Our previous research has indicated that land use composition surrounding hives impacts the “nutritional value” of bee breads (Donkersley et al. 2014). Other studies have tested variation in limited numbers of land use types over spatial gradients and how these impact pollinator foraging (Kaluza et al. 2016; Kämper et al. 2016). Although statistical analysis of land cover composition, as in our previous study (Donkersley et al. 2014), did not find statistically significant correlations between plant community composition (data not shown), this is likely due to the low level of spatiotemporal replication within the scope of this study, which has limited our confidence in statistical analysis. We may, however, make limited conclusions from the nature of the environment in our study site. Data from UK Countryside Survey Land Cover Map (Table S3; Carey et al. 2008) shows that within 3 km of these hives the most abundant landscape types are improved grasslands (occupying a total of 50% of all land in the study area) and urban environments (total 16%). Improved grasslands have been suggested to be of high nutritional value to pollinators nationally due to their relative high abundance of white clover nectar (Baude et al. 2016). Conversely, they also represent a low floral diversity (Tallowin et al. 2005); meaning that these environments, although they provide nectar and pollen when white clover is in flower, may lack the additional supplies of nutrients present in more diverse environments (Drescher et al. 2014).

Urban environments represent surprisingly abundant resources to pollinators, with high local biodiversity providing more nectar and pollen than surrounding natural environments (Kaluza et al. 2016; Somme et al. 2016). The results of this study potentially have implications for honey bee management, not only in terms of optimising hive location, but also for floral resource planting schemes in urban gardens and parks. Honey bees forage preferentially on certain plant species, and current recommendations for planting are based on these observations (Thompson et al. 2003). The prevalence of Himalayan balsam within this study suggests that the foraging requirements of honey bees in our study area are not being met by native forage species (Goverde et al. 2002; Pellissier et al. 2012), as they may not be present in sufficient abundance in the landscape. This hypothesis could be tested by a broader examination of the spatial relationship between foraging, food intake and land use composition (Kämper et al. 2016).

The results we present here indicate how diverse environments benefit their foragers by providing more optimal diets and compensating for the loss of individual forage species. Our study also adds to a growing body of data on pollinator forage in urban (and to a lesser extent pastoral and agricultural) environments, particularly in terms of non-native species. Future studies could relate these measures of hive pollen spectra directly to floral abundance and diversity in the surrounding landscape. This could allow for a more complete understanding of honey bee foraging behaviour related to nutritional reward, which could lead to recommendations on what to plant and conserve in agri-environmental schemes and urban bee projects.

## Electronic supplementary material

Below is the link to the electronic supplementary material.

Supplementary material 1 (DOC 23 kb)

Supplementary material 2 (DOC 33 kb)

Supplementary material 3 (DOC 102 kb)

Supplementary material 4 (DOC 29 kb)

Supplementary material 5 (DOC 73 kb)
