# Supplementary material for: Nutritional composition of honey bee food stores vary with floral composition
Source: Oecologia. 2017 Oct 14;185(4):749–61. doi: 10.1007/s00442-017-3968-3 (PMC5681600; doi:10.1007/s00442-017-3968-3)
Supplement: Supplementary file 1 — Supplementary material 1 (DOC 23 kb) [file 442_2017_3968_MOESM1_ESM.doc]

**Figure S1. Pollen genera abundance totalled across all bee bread samples.** The proportion of reads that account for each genus across the study; nineteen plant genus that accounted for >1% of the rarefied sequence reads, the remaining 73 rare genera that accounted for <1% of reads grouped into the “other” category.
