# Supplementary material for: Nutritional composition of honey bee food stores vary with floral composition
Source: Oecologia. 2017 Oct 14;185(4):749–61. doi: 10.1007/s00442-017-3968-3 (PMC5681600; doi:10.1007/s00442-017-3968-3)
Supplement: Supplementary file 2 — Supplementary material 2 (DOC 33 kb) [file 442_2017_3968_MOESM2_ESM.doc]

Figure S2. Correlations between sample and genus specific read counts with nutritional content determined from bee bread samples. NGS read counts have been log-transformed, and mean counts (±S.E.) have been calculated by grouping 1/6^th^ of the data. Hollow circles represent raw data, filled circles represent means.
