# Supplementary material for: Nutritional composition of honey bee food stores vary with floral composition
Source: Oecologia. 2017 Oct 14;185(4):749–61. doi: 10.1007/s00442-017-3968-3 (PMC5681600; doi:10.1007/s00442-017-3968-3)
Supplement: Supplementary file 3 — Supplementary material 3 (DOC 102 kb) [file 442_2017_3968_MOESM3_ESM.doc]

Table S1. Geographical locations of hives in the study site, internal relative position of cells segregated into Hive, Box and Frame, and mass of bee breads used for nutritional analysis and DNA extraction for sequencing (n = 51).

| Date | Apiary | Eastings | Northings | Hive | Box | Frame | Mass (mg) for nutrition | Mass (mg) for sequencing |
| --- | --- | --- | --- | --- | --- | --- | --- | --- |
| 12/05/2012 | AW | 525 | 346 | AW001 | i | a | 82.0 | 14.5 |
| 09/07/2012 | AW | 525 | 346 | AW001 | i | b | 139.2 | 24.6 |
| 24/08/2012 | AW | 525 | 346 | AW001 | i | b | 161.5 | 28.5 |
| 27/05/2012 | CM | 513 | 368 | CM001 | ii | c | 122.1 | 21.6 |
| 02/09/2012 | CM | 513 | 368 | CM002 | iii | d | 269.2 | 47.5 |
| 13/06/2012 | DR | 518 | 438 | DR001 | iv | e | 174.7 | 30.8 |
| 31/08/2012 | DRY | 855 | 307 | DRY01 | v | f | 57.5 | 10.1 |
| 31/08/2012 | DRY | 855 | 307 | DRY01 | v | f | 38.4 | 6.8 |
| 19/06/2012 | FR | 485 | 514 | FR001 | vi | g | 121.2 | 21.4 |
| 26/06/2012 | GC | 362 | 272 | GC001 | vii | h | 107.7 | 19.0 |
| 07/05/2012 | GH | 505 | 709 | GH001 | viii | i | 129.6 | 22.9 |
| 04/07/2012 | GH | 505 | 709 | GH002 | ix | j | 29.7 | 5.2 |
| 16/04/2012 | GT | 425 | 288 | GT001 | x | k | 105.1 | 18.5 |
| 16/04/2012 | GT | 425 | 288 | GT001 | xi | l | 187.7 | 33.1 |
| 12/06/2012 | GT | 425 | 288 | GT001 | xii | m | 204.6 | 36.1 |
| 02/08/2012 | GT | 425 | 288 | GT002 | xiii | n | 114.9 | 20.3 |
| 11/04/2012 | HJ | 468 | 662 | HJ001 | xiv | o | 187.7 | 33.1 |
| 08/06/2012 | HJ | 468 | 662 | HJ001 | xiv | o | 115.7 | 20.4 |
| 08/06/2012 | HJ | 468 | 662 | HJ001 | xiv | p | 147.6 | 26.1 |
| 08/06/2012 | HJ | 468 | 662 | HJ001 | xiv | p | 116.6 | 20.6 |
| 11/04/2012 | HJ | 468 | 662 | HJ001 | xv | q | 204.9 | 36.2 |
| 11/04/2012 | HJ | 468 | 662 | HJ001 | xv | r | 166.7 | 29.4 |
| 29/07/2012 | HJ | 468 | 662 | HJ002 | xvi | s | 66.9 | 11.8 |
| 13/07/2012 | JAH | 625 | 210 | JAH01 | xvii | t | 100.4 | 17.7 |
| 20/08/2012 | JAH | 625 | 210 | JAH01 | xvii | t | 108.9 | 19.2 |
| 25/07/2012 | JB | 547 | 361 | JB001 | xviii | w | 59.2 | 10.4 |
| 09/05/2012 | JH | 835 | 210 | JH001 | xix | x | 175.2 | 30.9 |
| 19/07/2012 | JH | 835 | 210 | JH001 | xix | x | 38.8 | 6.8 |
| 28/08/2012 | JH | 835 | 210 | JH001 | xix | y | 38.1 | 6.7 |
| 28/08/2012 | JH | 835 | 210 | JH001 | xix | y | 44.9 | 7.9 |
| 28/08/2012 | JH | 835 | 210 | JH001 | xix | x | 69.9 | 12.3 |
| 28/08/2012 | JH | 835 | 210 | JH001 | xix | x | 84.9 | 15.0 |
| 28/08/2012 | JH | 835 | 210 | JH001 | xix | y | 87.9 | 15.5 |
| 27/06/2012 | JM | 550 | 672 | JM002 | xx | z | 96.2 | 17.0 |
| 28/06/2012 | JM | 550 | 672 | JM002 | xx | z | 135.4 | 23.9 |
| 28/06/2012 | JM | 550 | 672 | JM002 | xx | aa | 78.0 | 13.8 |
| 28/06/2012 | JM | 550 | 672 | JM002 | xx | aa | 109.8 | 19.4 |
| 01/07/2012 | JP | 486 | 889 | JP001 | xxi | ab | 45.7 | 8.1 |
| 04/05/2012 | JP | 486 | 889 | JP001 | xxi | ac | 114.9 | 20.3 |
| 21/05/2012 | LW | 438 | 574 | LW001 | xxii | ad | 139.9 | 24.7 |
| 11/07/2012 | LW | 438 | 574 | LW001 | xxii | ad | 100.4 | 17.7 |
| 07/04/2012 | PC | 468 | 614 | PC001 | xxiii | ae | 34.9 | 6.2 |
| 23/07/2012 | PC | 468 | 614 | PC001 | xxiii | ae | 69.2 | 12.2 |
| 03/06/2012 | PC | 468 | 614 | PC001 | xxiv | af | 134.2 | 23.7 |
| 23/07/2012 | PC | 468 | 614 | PC002 | xxv | ag | 41.0 | 7.2 |
| 25/04/2012 | PM | 474 | 638 | PM001 | xxvi | ah | 87.6 | 15.5 |
| 02/05/2012 | PM | 492 | 594 | PM002 | xxvii | ai | 86.2 | 15.2 |
| 29/06/2012 | PM | 492 | 594 | PM004 | xxviii | aj | 141.4 | 24.9 |
| 29/06/2012 | PM | 492 | 594 | PM004 | xxviii | aj | 71.8 | 12.7 |
| 19/04/2012 | PS | 452 | 783 | PS001 | xxix | aq | 155.6 | 27.5 |
| 27/05/2012 | YC | 505 | 736 | YC001 | xxx | ar | 182.2 | 32.2 |
