# Supplementary material for: Nutritional composition of honey bee food stores vary with floral composition
Source: Oecologia. 2017 Oct 14;185(4):749–61. doi: 10.1007/s00442-017-3968-3 (PMC5681600; doi:10.1007/s00442-017-3968-3)
Supplement: Supplementary file 4 — Supplementary material 4 (DOC 29 kb) [file 442_2017_3968_MOESM4_ESM.doc]

**Table S2. Primers used in PCR reactions.** Italics indicate adaptor sequences including in primers for Illumina MiSeq workflow.

| Primer | Sequence (5`-3`) | Primer Target | Reference |
| --- | --- | --- | --- |
| S2F | 5` –*TCG TCG GCA GCG TCA GAT GTG TAT AAG AGA CAG* ATG CGA TAC TTG GTG TGA AT -3` | ITS2 region on 18S ribosomal gene | (Ch*en et a*l. 2010) |
| S3R | 5` -*GTC TCG TGG GCT CGG AGA TGT GTA TAA GAG ACA* GAC GCT TCT CCA GAC TAC AAT -3` | ITS2 region on 18S ribosomal gene | (Ch*en et a*l. 2010) |

**Nicotiana tabacum* chloroplast DNA numbering—corresponds to the positions in *N. tabacum* 18S chloroplast gene
