# Supplementary material for: Nutritional composition of honey bee food stores vary with floral composition
Source: Oecologia. 2017 Oct 14;185(4):749–61. doi: 10.1007/s00442-017-3968-3 (PMC5681600; doi:10.1007/s00442-017-3968-3)
Supplement: Supplementary file 5 — Supplementary material 5 (DOC 73 kb) [file 442_2017_3968_MOESM5_ESM.doc]

**Table S3.** Countryside Survey Land Cover Map (2007) % of each landscape cover type within a 3kM radius of all hives within this study. AGL = acid grassland; ARH = arable horticultural; BOG = bogs and marsh; BLW = broadleaf woodland; BUG = urban; CNW = coniferous woodland; DSH = shrub heathland; FWT = freshwater; IGL = improved grassland; INL = inland rock; NGL = neutral grassland; RGL = rough grassland; SWT = saltwater; SLS = litoral sands.

| Apiary | AGL | ARH | BOG | BLW | BUG | CNW | DSH | FWT | IGL | INL | LTS | NGL | RGL | SWT | SLS |
| --- | --- | --- | --- | --- | --- | --- | --- | --- | --- | --- | --- | --- | --- | --- | --- |
| PC | 0.0 | 1.2 | 0.0 | 2.0 | 42.0 | 0.4 | 0.0 | 1.1 | 35.0 | 0.2 | 9.4 | 0.2 | 4.2 | 4.2 | 0.1 |
| HJ | 0.0 | 1.4 | 0.0 | 1.1 | 18.5 | 0.4 | 0.0 | 0.4 | 36.2 | 0.0 | 33.5 | 0.1 | 4.3 | 3.9 | 0.2 |
| GT | 0.0 | 16.0 | 0.0 | 0.3 | 15.4 | 0.0 | 0.0 | 0.2 | 45.0 | 0.1 | 13.6 | 1.3 | 4.2 | 2.4 | 1.4 |
| PS | 0.0 | 4.2 | 0.0 | 21.3 | 6.0 | 1.1 | 0.6 | 0.8 | 23.8 | 0.7 | 31.9 | 2.9 | 4.2 | 1.8 | 0.5 |
| PMA | 0.0 | 1.2 | 0.0 | 3.6 | 41.2 | 0.6 | 0.0 | 1.2 | 41.0 | 0.2 | 4.6 | 0.2 | 4.3 | 1.9 | 0.1 |
| JP | 1.5 | 2.1 | 0.0 | 12.9 | 1.8 | 2.3 | 1.5 | 0.0 | 61.0 | 1.7 | 0.0 | 5.0 | 10.1 | 0.0 | 0.0 |
| GH | 0.2 | 2.9 | 0.4 | 9.5 | 13.5 | 1.0 | 0.7 | 2.3 | 51.4 | 2.7 | 7.0 | 0.3 | 8.1 | 0.0 | 0.1 |
| JH | 21.8 | 0.7 | 23.0 | 5.8 | 13.8 | 2.1 | 9.8 | 0.5 | 20.4 | 1.7 | 0.0 | 0.0 | 0.4 | 0.0 | 0.0 |
| AW | 0.0 | 6.1 | 0.0 | 3.0 | 29.0 | 0.9 | 0.0 | 0.1 | 56.5 | 0.8 | 0.0 | 3.1 | 0.5 | 0.0 | 0.0 |
| LW | 0.0 | 1.6 | 0.0 | 1.1 | 6.3 | 0.0 | 0.0 | 0.4 | 44.1 | 0.1 | 34.0 | 2.8 | 2.3 | 5.3 | 1.9 |
| CM | 0.0 | 7.1 | 0.0 | 1.5 | 9.1 | 0.4 | 0.0 | 0.2 | 74.8 | 0.7 | 0.0 | 4.4 | 1.6 | 0.0 | 0.0 |
| YC | 0.1 | 3.8 | 0.5 | 16.4 | 7.5 | 0.9 | 0.5 | 3.4 | 54.6 | 0.5 | 1.0 | 0.4 | 7.6 | 0.0 | 0.0 |
| DR | 0.0 | 3.6 | 0.0 | 7.6 | 6.6 | 1.1 | 0.0 | 0.2 | 73.1 | 0.0 | 0.0 | 3.5 | 4.3 | 0.0 | 0.0 |
| FR | 0.0 | 6.0 | 0.0 | 4.8 | 4.7 | 0.9 | 0.1 | 1.7 | 73.1 | 0.0 | 0.0 | 3.8 | 4.8 | 0.0 | 0.0 |
| PMB | 0.0 | 1.2 | 0.0 | 3.6 | 41.2 | 0.6 | 0.0 | 1.2 | 41.0 | 0.2 | 4.6 | 0.2 | 4.3 | 1.9 | 0.1 |
| JM | 0.4 | 1.6 | 0.0 | 8.0 | 1.7 | 0.6 | 0.4 | 1.6 | 76.6 | 0.0 | 0.0 | 4.1 | 5.0 | 0.0 | 0.0 |
| JAH | 2.8 | 2.7 | 25.2 | 8.5 | 4.7 | 1.8 | 3.0 | 1.3 | 40.7 | 0.1 | 0.0 | 0.0 | 9.1 | 0.0 | 0.0 |
| GC | 0.0 | 7.9 | 0.0 | 2.4 | 19.4 | 0.5 | 0.0 | 0.0 | 0.4 | 16.2 | 0.0 | 0.1 | 43.7 | 0.6 | 0.5 |
| JB | 0.0 | 5.7 | 0.0 | 2.5 | 12.9 | 0.7 | 0.0 | 0.0 | 71.7 | 0.0 | 0.0 | 4.6 | 1.9 | 0.0 | 0.0 |
| DRY | 15.8 | 1.0 | 0.0 | 7.7 | 24.7 | 0.9 | 8.8 | 0.2 | 37.5 | 0.4 | 0.0 | 0.0 | 3.1 | 0.0 | 0.0 |
